# Influence of the examination position and distension medium on the rectal sensory test in patients with functional constipation

**DOI:** 10.1186/s12876-024-03309-5

**Published:** 2024-07-29

**Authors:** Chang-Fang Xiao, Yi-Fan Li, Yang-Yang Sun, Ling-Yun Meng, Jing-Wen Wu, Min Wang, Yong-Qing Cao, Chen Wang, Yi-Bo Yao

**Affiliations:** Department of Anorectal Surgery, Longhua Hospital, Shanghai University of Traditional Chinese Medicine, Shanghai, 200030 China

**Keywords:** Functional constipation, Rectal hyposensitivity, Body position, Distension medium, Sensory threshold measurement

## Abstract

**Purpose:**

To evaluate the impact of two different parameters (body position and distension medium) on the rectal sensory test in patients with functional constipation and provide data support for the development of standardized operating procedures in clinical practice.

**Methods:**

Based on a single-center process of the rectal sensory test, 39 patients with functional constipation were recruited for rectal sensory test under different body positions and distension mediums.

**Results:**

Among the items of the Constipation Scoring System, the score of frequency of bowel movements showed a negative correlation with the first constant sensation volume (*r* = -0.323, *P* = 0.045). Conversely, the score of painful evacuation effort showed a positive correlation with the desire to defecate volume (*r* = 0.343, *P* = 0.033). There was a statistically significant difference in the first constant sensation volume (when the distension medium was gas) measured in different body positions (left lateral position, sitting position, squatting position), and the data measured in the squatting position were significantly higher than those in left lateral position (*P* < 0.05). In terms of research on distension medium, it was found that the first constant sensation volume measured in the squatting position (when the distension medium was water) was significantly lower than that of gas (*P* < 0.05).

**Conclusion:**

For patients with functional constipation, there are differences in the results of rectal sensory tests between body positions and distension mediums. When conducting multicenter studies, it is necessary to unify the standard operating procedure (SOP) for operational details to ensure consistency and reliability of the test results.

## Introduction

Functional constipation is a common clinical disease characterized by reduced bowel movements, dry stools, and difficulty in defecation [1]. With the increasing trend of population aging, its incidence rate is increasing year by year. The latest epidemiological data suggest that the global incidence of functional constipation is 11.6–32.3% [2–5]. However, due to issues such as poor clinical treatment efficacy, drug abuse, and susceptibility to recurrence, the prevention and clinical treatment of functional constipation have become common focuses of attention worldwide [6].

With in-depth research on its pathogenesis, the important role of rectal sensation in defecation has gradually been recognized, and rectal hyposensitivity (RH) is considered an important cause of functional constipation [7]. RH was first described in 1951 in patients who had undergone parasympathetic block prior to surgery [8]. RH is presented as a diminished perception of rectal distension and defined as an elevation of 1 or more of the 3 sensory thresholds (FCSV, DDV and MTV) in most studies [9]. Abnormal visceral sensitivity is widely considered important in the development of functional bowel disorders; however, the role of visceral insensitivity remains relatively unexplored compared with the study of visceral hypersensitivity in the past [10]. Normal rectal sensory function is crucial for the defecation process [11]. 48% of patients with constipation have impaired rectal sensation as the only abnormality during physiological examinations [10]. RH may participate in the occurrence and development of constipation through various mechanisms, leading to symptoms such as weakening of the desire to defecate, prolonged bowel movements, and incomplete rectal emptying [12, 13]: (1) stool retention: the lack of desire to defecate can lead to retention of feces, and the accumulation of more dry and hard feces will make it more difficult to discharge (and likely cause further dilation of the rectum); (2) reduced rectal contractility: when RH is accompanied by rectal dilation, it can reduce rectal contractility and affect bowel movements; (3) primary colonic peristalsis disorder: one-third of RH patients have been confirmed to have delayed colonic transmission, which may reflect a primary colonic peristalsis disorder.

An anorectal physiology analysis is conducted for patients with symptoms of anorectal dysfunction to identify potential pathophysiological mechanisms. Measurement of the rectal sensory threshold is one of the steps in the diagnosis of functional bowel dysfunction. The International Anorectal Physiology Working Group (IAPWG) published a standardized measurement protocol for anorectal function testing in 2020, but the details of sensory threshold measurement still need to be further clarified [14]. The inconsistency in specific measurement details has led to heterogeneity in various studies [15, 16]. There are many influencing factors for this method, which leads to heterogeneity among various studies. The influencing factors include [14, 17–19] (1)the type of distension: ramp distension and phasic distension; (2)distension medium: gas and water; (3)inflation rate: 10 ~ 100 mL/mins; (4)the distance of the balloon from the anal margin; (5)Examination position. The traditional measurement method is the left lateral position, which is not in line with human physiological defecation, and its data may affect the judgment of clinical physicians [20, 21]. Especially when required to perform defecation in lateral position, some patients may exhibit abnormal contraction or incomplete relaxation of anal pressure due to their inability to adapt to the lateral lying position bowel habits [22]. Different stool types can affect bowel movement [11, 23]. Therefore, it is necessary for pelvic floor surgeons to study and formulate a unified standard. Therefore, this single-center study was conducted to analyze the impact of different test parameters (body position and expanding medium) on rectal sensory tests in patients with functional constipation and to explore appropriate detection processes and technical parameters.

## Patients and methods

This study recruited patients with functional constipation from the Anorectal Clinic from June 2021 to February 2022 to perform rectal sensory tests with different positions and distension mediums. The patient met the Roman IV diagnostic criteria for functional constipation [24]. Exclusion criteria: (1) patients with acute perianal disease accompanied by infection or bleeding symptoms. (2) Patients with rectal prolapse, rectal protrusion, and other organ prolapse. (3) patients who have undergone surgery for benign perianal diseases. (4) patients with diabetes mellitus accompanied by poorly controlled blood sugar or diabetic neuropathy. (5) patients with severe mental illness who could not cooperate with the examination; (6) patients with drug-induced constipation and secondary constipation with clear etiology, such as Parkinson’s disease, hypothyroidism, and spinal cord injury.

### Procedures

**Fig. 1:**
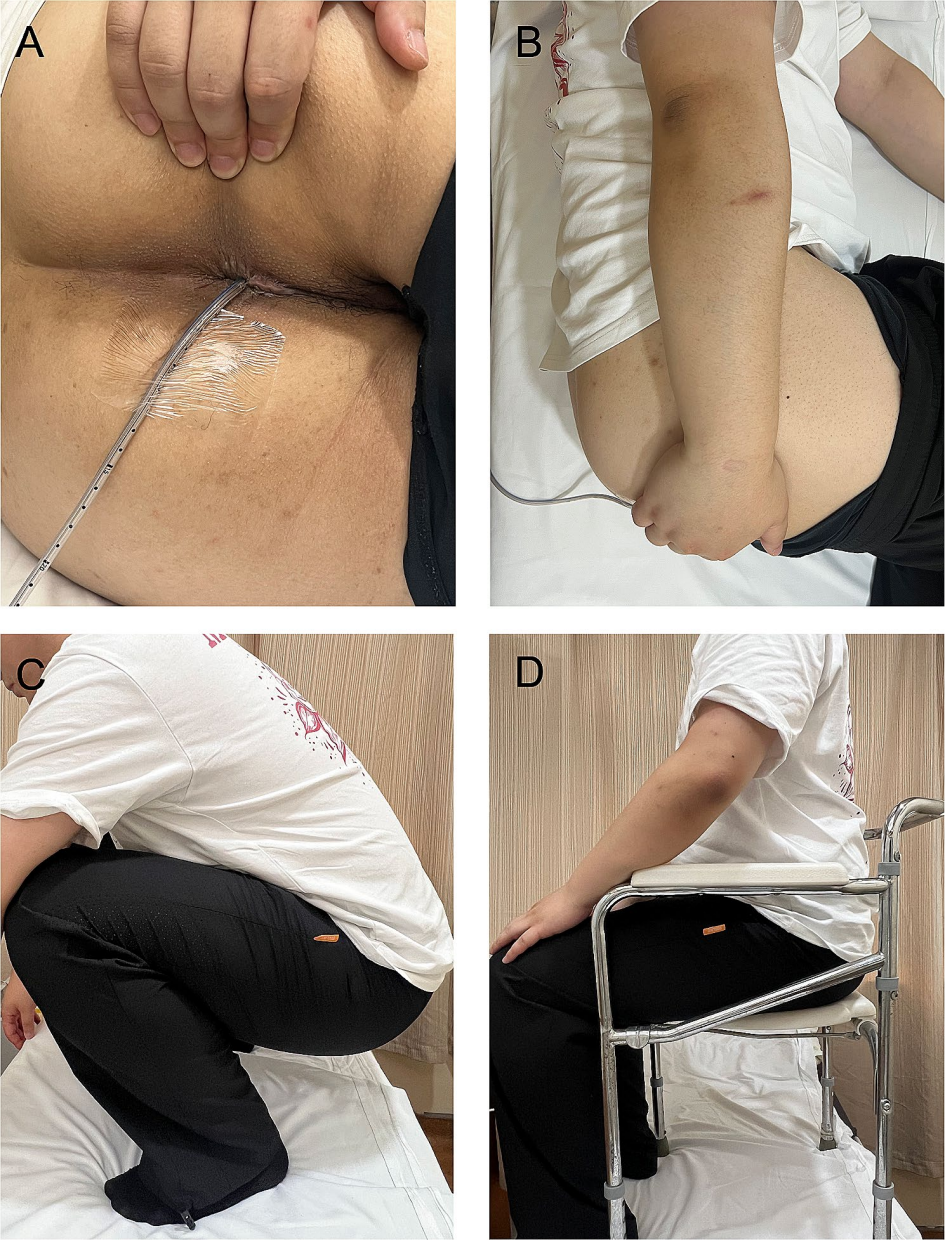
The schematic diagram of the fixing the catheter and the position of the patient for examination. (**A**) a piece of transparent adhesive tape was used to fix the catheter to the left perianal skin 1–2 cm from the anus; (**B**) left lateral position; (**C**) squatting position; (**C**) sitting position

The rectal sensory test, including the first constant sensation volume (FCSV), desire to defecate volume (DDV), and maximum tolerated volume (MTV), was performed using a catheter and balloon equipped with an anorectal motility analyzer (Triton, Leibri, Canada). Two hours before the test, 20 ml of a glycerin enema was used to empty feces from the rectum, and the given patient was informed of the meaning of FCSV, DDV and MTV [14]. The specific procedures of anorectal pressure test were as follows: rest, squeeze, long (endurance) squeeze, cough, and push. The whole process should be explained in detail to the patient [14]. First, the pressure-measurement tube was put into the anus and tell the patient to relax and remain quiet to avoid movement artifact, which lasted for 60 s. Secondly, ask the patient to contract his anus for 5 s 3times separated by a 30-s between‐maneuver recovery interval. Thirdly, the patient was then asked to perform a sustained contraction for 30 s and the pressure was recordedrecords the anal pressure during sustained voluntary effort over 30 s. A single endurance squeeze is performed followed by a 60‐s between‐maneuver recovery interval. Fourthly, the patient was asked to perform the coughing maneuver twice, 30s apart, and the change in pressure was observed. Fifthly, the patient was asked to perform three simulated defecation movements, each 15s, with an interval of 30s. Body position and distension parameters were fixed in the study for evaluation purposes, with the specific parameters as follows: (1) distension method: continuous balloon distension method was used to inject gas or water at a rate of 1 mL/s; (2) distension medium: gas and water (at room temperature) for patients separately; (3) distension speed: injection with a handheld syringe at a speed of 1 mL/s; (4) distance between the bottom of the balloon and the anal margin: 5 cm; (5) position: left lateral position, squatting position, and sitting position separately, with an interval of 10 min between each measurement position. Considering the safety of detecting MTV (balloon bursting, balloon unable to pump water), an MTV warning line of 180 ml was set in this study (if it was over 180 ml, water injection was stopped); (6) fixation of the balloon position: a piece of transparent adhesive tape was used to fix the catheter to the left perianal skin 1–2 cm from the anus to keep the tube somewhat movable but not easily dislodged during the test. If the catheter became dislodged or displaced during the three-position test, the catheter had to be reattached and the test repeated. All patients were tested by the same therapist and placed in the left lateral position for balloon insertion using the same test sequence of left lateral position (gas), left lateral position (water), sitting position (gas), sitting position (water), squatting position (gas) and squatting position (water). The schematic diagram shows the method of fixing the catheter and the position of the patient for examination (Fig. 1).

### Statistical analysis

Quantitative variables are presented herein as the mean ± SD. Qualitative variables are presented as counts (percentages). For the comparison of measurement data between the two groups, the normality test was first conducted, the *t* test was used for the normal distribution and the homogeneity of variance, and the Mann‒Whitney U nonparametric test was used for the nonnormal distribution. One-way ANOVA was adopted if the measurement data between multiple groups conformed to a normal distribution. If it did not conform to a normal distribution, the Kruskal‒Wallis H test was adopted. The chi-squared test was used to compare the rates of multiple groups. Relationships between the constipation scale and rectal sensory function (FCSV, DDV and MTV) were assessed by Spearman’s correlation coefficient. The difference was statistically significant at *P* < 0.05.

## Results

### Population

A total of 39 patients with functional constipation were included in the study and analysis of the impact of different positions and distension mediums on rectal sensory test. Among them, the proportion of female patients is higher than that of males, with 69.23% being female and 30.77% being male; The average age was 53.85 ± 14.19 years old, and the overall research subjects were mainly middle-aged and elderly; The average BMI was 22.70 ± 3.00 kg/m [2], which is generally in line with the normal body mass index; The rectal resting pressure, anal resting pressure, functional anal canal length, and maximum anal squeeze pressure in the left lateral position were all within the normal range evaluated by our unit. The constipation scale for enrolled patients, including the Constipation Scoring System (CSS), the Patient Assessment of Constipation Symptoms (PAC-SYM), and the Patient assessment of constipation quality of life questionnaire (PAC-QoL) are shown in Table 1.

**Table 1:** Patient characteristics

Characteristics	Value
Sex(n)	39
Male(n, %)	12, 30.77%
Female(n, %)	27, 69.23%
BMI(kg/m [2], $\bar x \pm s$)	22.70 ± 2.98
Age(y/o, $\bar x \pm s$)	53.85 ± 14.19
Rectum resting pressure*(mmHg, $\bar x \pm s$)	10.41 ± 4.81
Anal resting pressure*(mmHg, $\bar x \pm s$)	52.59 ± 13.43
Functional anal canal length*(cm, $\bar x \pm s$)	2.15 ± 0.26
Maximum anal squeeze pressure*(mmHg, $\bar x \pm s$)	167.54 ± 39.99
CCS(scores, $\bar x \pm s$)	15.18 ± 4.93
CCS-frequency of bowel movements(scores, $\bar x \pm s$)	0.31 ± 0.61
CCS-painful evacuation effort(scores, $\bar x \pm s$)	3.23 ± 1.18
CCS-incomplete evacuation(scores, $\bar x \pm s$)	3.31 ± 1.18
CCS-abdominal pain(scores, $\bar x \pm s$)	1.87 ± 1.53
CCS-length of time per attempt(scores, $\bar x \pm s$)	1.97 ± 1.44
CCS-assistance for evacuation(scores, $\bar x \pm s$)	1.18 ± 0.85
CCS-unsuccessful attempts for evacuation per 24 h(scores, $\bar x \pm s$)	0.90 ± 0.82
CCS-duration of constipation(scores, $\bar x \pm s$)	2.41 ± 1.27
PAC-SYM(scores, $\bar x \pm s$)	19.62 ± 5.19
PAC-QOL(scores, $\bar x \pm s$)	66.87 ± 19.92

*Tested in the left lateral position

### Relationships between constipation scale and rectal sensory function

**Fig. 2:**
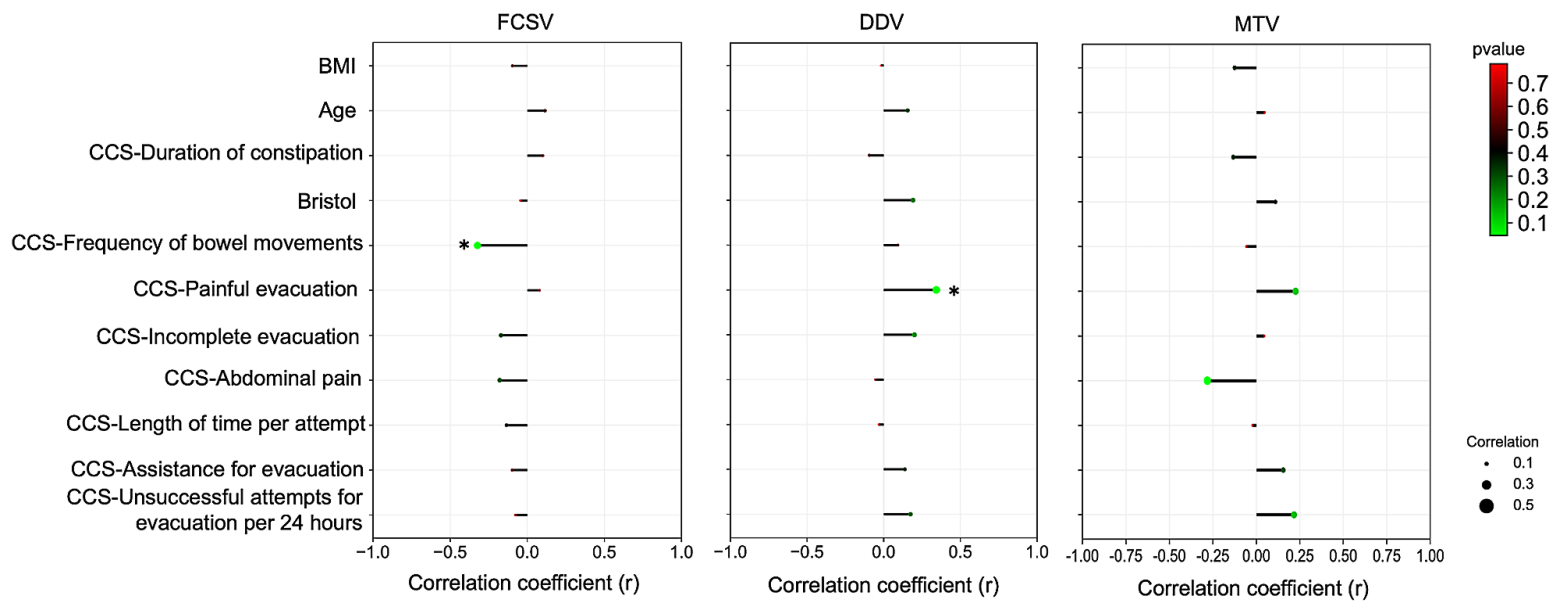
Relationships between the constipation scale and rectal sensory function. Abbreviations: * indicates *p* < 0.05, FCSV: first constant sensation volume, DDV: desire to defecate volume, MTV: maximum tolerated volume. FCSV, DDV and MTV were tested in the left lateral position using gas as the distension medium

There was no correlation between BMI, age and rectal sensory function. Among the items of the CSS, the score of frequency of bowel movements showed a negative correlation with FCSV (*r* = -0.323, *P* = 0.045). In contrast, the score of painful evacuation effort showed a positive correlation with DDV (*r* = 0.343, *P* = 0.033) (see Fig. 2).

### Influence of distension medium on rectal sensory test

FCSV, DDV and MTV were evaluated by different distension media (water, gas) in the left lateral position and sitting position, and it was found that the differences were not statistically significant. The only significant difference was DDV between water or gas was in squatting (*P* = 0.017), indicating that in the squatting state, the sensitivity of the water evaluation is higher than that of the gas evaluation (see Table 2).

**Table 2:** Influence of the distension medium on the rectal sensory test

Position	Rectal sensory test	medium	Value	*P*
Left lateral position	FCSV(mL, $\bar x \pm s$)	Gas	35.26 ± 20.77	0.429
		Water	40.21 ± 21.83	
	DDV(mL, $\bar x \pm s$)	Gas	87.95 ± 31.03	0.182
		Water	81.28 ± 33.48	
	MTV(>180mL, %)	Gas	17, 43.60%	0.153
		Water	10, 25.64%	
Sitting position	FCSV(mL, $\bar x \pm s$)	Gas	46.79 ± 23.47	0.702
		Water	46.15 ± 25.79	
	DDV(mL, $\bar x \pm s$)	Gas	94.62 ± 32.39	0.074
		Water	83.97 ± 32.55	
	MTV(>180mL, %)	Gas	10, 25.64%	0.250
		Water	5, 12.82%	
Squatting position	FCSV(mL, $\bar x \pm s$)	Gas	53.59 ± 30.78	0.405
		Water	45.64 ± 22.42	
	DDV(mL, $\bar x \pm s$)	Gas	98.97 ± 36.00	0.017
		Water	82.82 ± 36.78	
	MTV(>180mL, %)	Gas	13, 33.33%	0.195
		Water	7, 17.95%	

### Influence of examination position on rectal sensory test

When gas was used as the distension medium, the difference in the FCSV between different body positions (left lateral, sitting, squatting) was statistically significant (*P* = 0.007). Further multiple comparisons were used, and significance values were adjusted using the Bonferroni correction method. There was a significant difference between the left lateral and squatting positions (*P* = 0.008). There was no statistically significant difference between the DDV and the MTV in different body positions in this condition. When water was used as the measuring medium for distension, there was no statistically significant difference in the FCS, DDV and MTV between different body positions (see Table 3).

**Table 3:** Influence of the examination position on the rectal sensory test

Distension medium	Rectal sensory test	Position	Value	*P*
Gas	FCSV(mL, $\bar x \pm s$)	Left lateral	35.26 ± 20.77	0.007
		Sitting	46.79 ± 23.47	
		Squatting	53.59 ± 30.78*	
	DDV(mL, $\bar x \pm s$)	Left lateral	87.95 ± 31.03	0.393
		Sitting	94.62 ± 32.39	
		Squatting	98.97 ± 36.00	
	MTV(>180 ml, %)	Left lateral	17, 43.60%	0.244
		Sitting	10, 25.64%	
		Squatting	13, 33.33%	
Water	FCSV(mL, $\bar x \pm s$)	Left lateral	40.21 ± 21.83	0.368
		Sitting	46.15 ± 25.79	
		Squatting	45.64 ± 22.42	
	DDV(mL, $\bar x \pm s$)	Left lateral	81.28 ± 33.48	0.725
		Sitting	83.97 ± 32.55	
		Squatting	82.82 ± 36.78	
	MTV(>180 ml, %)	Left lateral	10, 25.64%	0.346
		Sitting	5, 12.82%	
		Squatting	7, 17.95%	

*: Compared with the left lateral position, the difference was statistically significant (*P* < 0.05)

## Discussion

The exact cause of RH is not yet clear, but there is evidence to suggest that constipation, long-term inhibition of defecation, pelvic nerve injury, spinal trauma, perianal surgery, etc. may be related factors [25]. With the deepening of research on functional constipation, the importance of RH has been gradually recognized in functional constipation. In the research on the correlation between functional constipation and RH, a cross-sectional study (including 218 cases) found that 56% of functional constipation cases were accompanied by RH [9]. The rectal sensory function is performed in a left lateral position instead of a seated position, however the latter is the physiological position for defecation [21]. At the same time, the sensation of defecation is related to the size and weight of the feces [26, 27]. Therefore, this study designed to use different positions and distension mediums for rectal sensation measurement, to evaluate whether there are differences and whether the results can be mutually substituted in clinical practice.

At present, the main method for measuring rectal sensory function is volume measurement using rectal dilation, which is widely used in clinical practice [10, 19]. Balloon distension is currently the most commonly used evaluation method for rectal sensory function, which uses a handheld syringe to slowly inject gas/water to expand the balloon and reflects the sensitivity of the rectum based on the volume of injection [18]. Simple balloon distension appears satisfactory for the initial assessment of rectal sensory function and will identify patients with elevated sensory threshold volumes [28].

On the basis of simple balloon distension, barostat distension was invented [19]. A barostat is an electromechanical device that uses a computer-controlled pressure regulator and a highly flexible polyethylene bag to provide equal pressure rectal dilation for more accurate measurement [28–30]. Barostat can provide information on the biomechanical properties of the intestinal wall, but it has not yet been widely used in clinical practice due to the long operation time (60–90 min) and high cost [31]. In addition to the volumetry-based methods, there are currently relevant studies using a bipolar ring electrode supplying a constant current to test the electrical sensitivity of rectal mucosa. Researchers believe that electrical testing avoides the variables inherent in balloon distension and is well tolerated, accurately quantifiable, and reproducible [32]. The heat stimulation method has also been explored as a method for evaluating rectal sensation. The median rectal heat threshold was found to be similar in males (median, 47 degrees C; range, 44–50 degrees C) compared with females (median, 45 degrees C; range, 43–50 degrees C; *P* > 0.05) and there was a high degree of repeatability with rectal heat and balloon distension thresholds (MTV, *r* = 0.8; *P* < 0.001, measured with balloon distension) [33]. Although the rectum is sensitive to electrical and thermal stimulation, mechanical distention is the most reliable and physiologic stimulus for the assessment of sensation [19]. Electrical stimulation and heat stimulation are considered to bypass the end organ receptor, which can bypass the mucosal receptor and directly depolarize the free nerve ending, directly stimulate the surrounding neuronal axons, and cannot reflect the situation of rectal mucosal receptor [12]. At the same time, electrical stimulation and heat stimulation cannot simulate normal bowel stimulation. There is currently no consensus on whether the nonmechanical distension method represented by electrical stimulation and heat stimulation can replace volumetry. Therefore, it is currently limited to research and has not been clinically applied [19].

There are currently studies measuring the differences between the left lateral position, the squatting position, sitting position, and lithotomy position, but the results are heterogeneous (see Table 4). The method of using gas as a distension medium is widely used in clinical practice [10]. However, although there are relevant studies on whether there are differences between the distension medium of gases and other media, the conclusions are inconsistent (see Table 4). During rectal sensitivity testing using gas and water as distension media in 12 normal males, it was found that there was no difference between gas and water [17]. For patients with prolapsed hemorrhoids, the FCSV of gas is significantly lower than that of water in the left lateral position and the MTV of gas is significantly higher than that of water in the sitting position [34].

**Table 4:** Studies on the influence of body position on rectal sensory test

Author, Year	Object	Case number	Position	Distension medium	Conclusion
Kadam-Halani PK, 2020 [35]	Woman	21	Left lateral position; Lithotomy position	Gas	FCSV, DDV and MTV measured in the lithotomy position are higher than those in the left lateral position.
Zhang CX, 2020 [22]	Patients with constipation/bulging or pain;	Constipation :46; bulging or pain: 20;	Semirecumbent lithotomy, left lateral position	Gas	The MTV was higher with the left lateral position than with the semirecumbent lithotomy position (*P* < 0.05)
Li YH, 2017 [36]	Patients with constipation	20	Left lateral position; sitting position	Gas	No difference between the FCSV, DDV and MTV
Wu JY, 2015 [37]	Patients with constipation and healthy volunteers	Constipation :20; healthy volunteers: 20;	Left lateral position; sitting position	Gas	No difference between the FCSV, DDV and MTV
Wang C, 2013 [34]	Patients with prolapsed hemorrhoids	20	Left lateral position; sitting position; squatting position	Gas and water	In the left lateral position, the FCSV of gas was significantly lower than that of water; In the sitting position, the MTV of gas was significantly higher than that of water;
Sun W M, 1990 [17]	Normal male	12	Left lateral position	Gas and water	No difference between the DDV.

Sorted by year of publication

Based on the above literature research results, it was found that there is heterogeneity in the research results of rectal sensory testing between different body positions and different distension medium, which may be due to the different research subjects and specific operating parameters used. Therefore, this study fixed the relevant operating parameters for rectal sensory testing for patients with functional constipation. In this study, it was found that there was a statistically significant difference in the FCSV (distension medium: gas) among different body positions (left lateral position, sitting position, squatting position), and the data measured in the squatting position were significantly higher than those in the left lateral position (*P* < 0.05). The difference may be due to relaxation of the puborectal and pelvic floor muscles during squatting, opening of the right angle of the anus, straightening of the angle of the rectum and anal canal, and anatomical changes affecting the patient’s perception of stimuli [11, 38, 39]. At the same time, the patient’s attention and sensory abilities may be distracted by maintaining the body balance and feeling uncomfortable in the legs when using a squatting position, causing an increase in the sensory threshold value. A thorough evaluation of the patient’s anorectal function is important, especially in terms of patient referrals to different treatment centers and pre- and post-treatment assessments. We found that the data measured in the sitting position were more stable when either gas or water was the medium, and were not significantly different from the other positions. Water was also more stable than gas. At the same time, considering that the current defecation habits of people are mainly in the sitting position and that feces have a certain weight, it is recommended to use the sitting position and water as the medium for the measurements, taking into account the data of this study.

To explore the relationship between symptom scales and rectal sensory thresholds, we correlated all 8 items of CCS with sensory thresholds and found that FCSV were negatively correlated with the score of frequency of bowel movements (*r* = -0.323, *P* = 0.045) and incomplete evacuation (*r* = -0.170, *P* = 0.300). This suggests that there may be a relative increase in rectal sensitivity in this group of patients with fewer bowel movements. On further analysis of the relationship between CCS total score and rectal sensitivity, no significant correlation was found. We analysed that the each item in the CCS scale do not represent the overall change in constipation symptoms. However, due to our small sample size in this case, there may be a bias in the data. Therefore, further expansion of the sample size is needed in future studies.

Anorectal manometry has been widely utilized in clinical treatment and assessment. Several studies have indicated significant variations in anorectal manometry results based on different positions. This study aims to compare and analyze the impact of different examination positions and dilatation media on rectal sensory detection in patients with functional constipation, in order to recommend more suitable examination positions and testing media for clinical use. The goal is to fully capture anorectal changes under physiological conditions, provide more precise data for clinical practice, and offer data support for future studies with larger samples. The sample size of this study was small, so the data may be statistically biased. Patients were not given comprehensive constipation-related tests, including colonic transit time and whole colon manometry. Patients were also not stratified and analysed according to severity or subtype of constipation. Therefore, there is a need for subsequent studies to include larger sample sizes and more detailed and comprehensive assessments to conduct in-depth research.

## Conclusion

Based on the results of this study, there are differences in the results of sensory testing between body positions and distension mediums. It is necessary to use the commonly used defecation posture of patients for rectal sensation testing rather than collecting data from the left lateral position alone. Taking into account the data from this study, it is recommended to use the sitting position and water as the medium for the measurements. However, due to the small sample size of this study, there may be bias in the data. When formulating standard values, each center needs to pay attention to the setting of details such as the body position and the distension medium. When conducting multicenter studies, it is necessary to unify the standard operating procedure (SOP) settings for operational details to ensure the consistency of the study.

## Data Availability

Data can be made available from the Corresponding Author on reasonable request.

## Abbreviations

SOP: Standard operating procedure
RH: Rectal hyposensitivity
BMI: Body Mass Index
CSS: Constipation Scoring System
DDV: Desire to defecate volume
FCSV: First constant sensation volume
IAPWG: International Anorectal Physiology Working Group
MTV: Maximum tolerated volume
PAC-QoL: Patient assessment of constipation quality of life questionnaire
PAC-SYM: Patient Assessment of Constipation Symptoms
